# Vitamin A Deficiency in Children With Autism Spectrum Disorder

**DOI:** 10.7759/cureus.77129

**Published:** 2025-01-08

**Authors:** Yuki Ozawa, Akiko Hikoya, Nobutaka Tachibana, Miwa Komori, Tokiko Fukuda, Hidetoshi Ishigaki, Takuya Hiraide, Miho Sato

**Affiliations:** 1 Ophthalmology, Hamamatsu University School of Medicine, Hamamatsu, JPN; 2 Ophthalmology, Japanese Red Cross Shizuoka Hospital, Shizuoka, JPN; 3 Hamamatsu Child Health and Development Medicine, Hamamatsu University School of Medicine, Hamamatsu, JPN; 4 Pediatrics, Hamamatsu University School of Medicine, Hamamatsu, JPN

**Keywords:** autism spectrum disorder, children, corneal ulcer, eating disorders, keratomalacia, night blindness, optic neuropathy, vitamin a deficiency, xerophthalmia, xerosis

## Abstract

Vitamin A deficiency (VAD) can manifest as xerophthalmia, a spectrum of eye conditions ranging from night blindness to severe outcomes like keratomalacia and corneal scarring.

We report two cases of a seven-year-old girl and a 13-year-old boy in Japan with xerophthalmia caused by VAD associated with autism spectrum disorder (ASD). The first case demonstrates severe complications of prolonged VAD, including optic disc edema and irreversible corneal damage, which then results in blindness. The second case identified and treated earlier in the stage of corneal xerosis shows favorable prognosis. These two cases illustrate the risk of VAD among children with ASD and the importance of timely intervention to prevent permanent vision loss.

Because of the prevalence of ASD, awareness of VAD as a potential health problem needs to be raised. Early detection, proper treatment, and dietary guidance are crucial in managing VAD. Ophthalmologists must be vigilant for signs of VAD in children with ASD, including difficulty opening eyelids, to prevent irreversible visual loss.

## Introduction

Vitamin A deficiency (VAD) is a significant public health issue, causing substantial illness and death due to increased susceptibility to common childhood infections. It is also the world’s leading preventable cause of childhood blindness [1-3]. VAD can manifest as xerophthalmia, a spectrum of eye conditions ranging from night blindness to severe outcomes like keratomalacia and corneal scarring [1]. Xerophthalmia remains a major public health concern, particularly in Southeast Asia and Africa, where it is estimated to cause blindness in 250,000-500,000 children annually [2,3]. When traditionally considered a problem in developing countries, recent years have seen increasing reports of VAD among children with autism spectrum disorder (ASD) in developed nations [4,5].

This report details two cases of VAD associated with ASD, both resulting from unbalanced diets in Japan with different courses.

## Case presentation

Case 1

A seven-year-old girl with ASD was referred to our clinic due to corneal opacity and difficulty opening both eyes. Three months prior, she began rubbing her eyes excessively. Her medical history included epilepsy diagnosed at age three and hypothyroidism diagnosed at age six. She received regular checkups at a pediatrician's office. Two months before presentation, she developed a pubic ulcer. For several years, she had exhibited eating disorders, limiting her diet primarily to white rice and rice crackers. While she previously ate fish, she recently stopped consuming meat, milk, eggs, or vegetables. Additionally, she did not take any supplements. Her food intake further decreased due to pain from the pubic ulcer.

**Figure 1:**
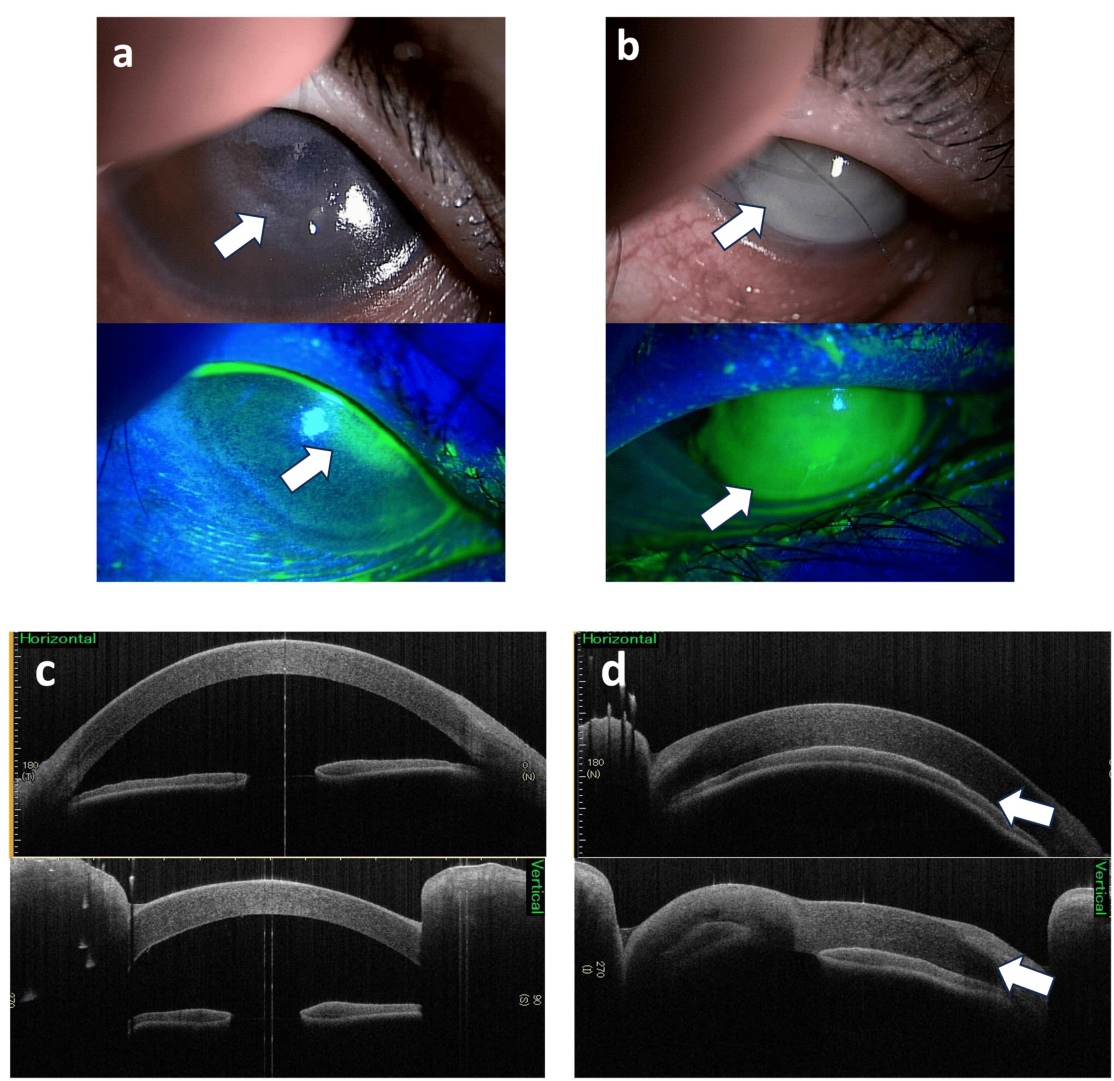
Slit-lamp examination revealed marked dryness and folds in the bilateral ocular conjunctiva associated with xerophthalmia. The figures presented show fluorescein staining of the eyes. a. The right cornea exhibited ulceration and dense superficial punctate keratopathy. b. The left cornea displayed softening and edematous opacity with the loss of the corneal epithelial barrier. c. The right eye appeared normal. d. The left anterior chamber was flat, and the cornea was edematous and thickened.

**Figure 2:**
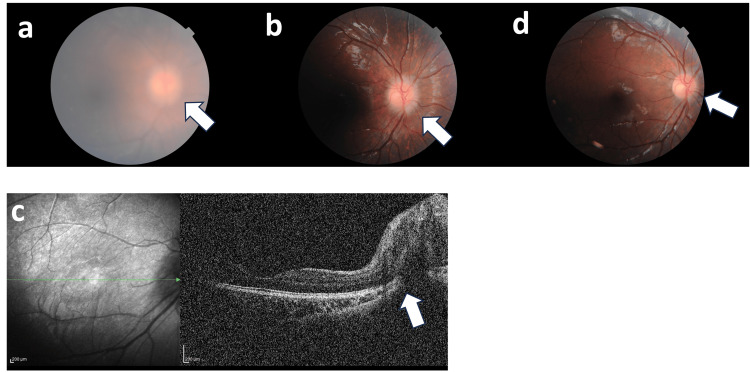
Color fundus photographs and OCT a. On the day of the initial examination, the fundus photograph revealed optic disc swelling in the right eye. Photography of the left eye was impossible due to severe corneal opacity. b, c. Day 15 of treatment. The fundus became translucent, but optic disc swelling remained prominent. OCT demonstrated obvious disc swelling. d. Day 35 of treatment. Swelling of the disc was reduced. OCT: Optical coherence tomography

**Figure 3:**
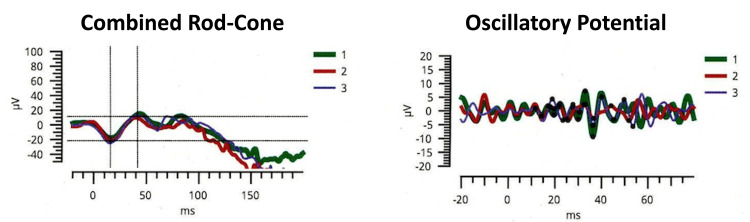
Skin electrode electroretinogram in the right eye before treatment Full-field electroretinography of the right eye reveals a reduction in combined rod-cone and oscillatory potential.  The left eye was unmeasurable due to noise.

**Figure 4:**
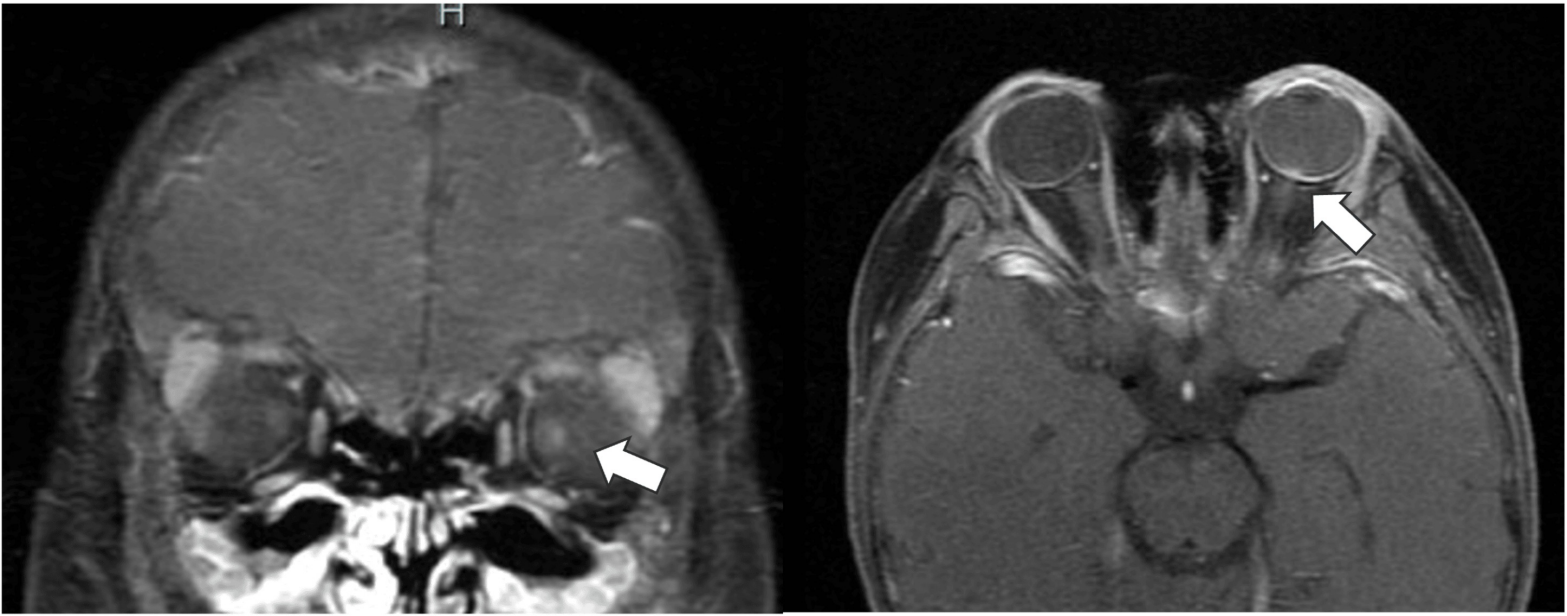
Contrast-enhanced MRI of the head Contrast-enhanced MRI after admission showed a mild contrast-enhanced effect in the left peripapillary and anterior optic nerve. There was no hyperostotic change.

On examination, the girl appeared short and underweight for her age, measuring 98 cm in height (-4.47 standard deviation: SD) and weighing 12.95 kg (-5.35 SD). Both the ocular conjunctiva and cornea exhibited dryness and keratosis. The right cornea lacked its epithelial layer, and the left cornea displayed ulceration and opacity (Figures 1a, 1b). Anterior segment optical coherence tomography (OCT) revealed a loss of the anterior chamber in the left eye (Figures 1c, 1d). Color fundus photography showed papilledema in the right eye (Figure 2a). The left eye was obscured by corneal opacity. A skin electrode electroretinogram (SEE) showed attenuated responses (a, b, and OP waves) in the right eye, with undetected responses in the left eye (Figure 3). Contrast-enhanced magnetic resonance imaging (MRI) of the head showed mild enhancement in the left peripapillary and anterior optic nerves, but no bone overgrowth (Figure 4). Her best-corrected visual acuity (BCVA) was 0.06 with decimal visual acuity in the right eye and light perception in the left eye.

Based on medical history and clinical findings, VAD was suspected. A blood test confirmed low serum vitamin A levels (below detection limit: ≦5 IU/dL; normal: 97-316 IU/dL) along with deficiencies in other nutrients: prealbumin, 10.8 mg/dL (normal: 22-40 mg/dL); retinol binding protein, 0.6 mg/dL (normal: 1.9-4.6 mg/dL); folate, 2.9 ng/mL (normal: 5-12 ng/mL); vitamin B12, 150μg/dL (normal: 180-914μg/dL); 25-hydroxy-vitamin D, 8.4 ng/mL (normal: >30ng/mL); zinc, 44 μg/dL (normal: 80-130μg/dL); and selenium, undetectable (<2.0 μg/dL, normal: 7.7-14.8μg/dL). The patient was diagnosed with xerophthalmia and keratomalacia due to VAD. She received oral and intravenous multivitamins (containing 5,500 IU of vitamin A). Additionally, tube feeding (containing approximately 630 IU vitamin A daily) and zinc acetate supplementation were initiated.

Approximately ten days after treatment began, the conjunctiva showed increased moisture, and the cornea of the right eye became transparent. The BCVA in the right eye improved to 0.5. Despite only light perception in the left eye, the ulcer shrank, and the anterior chamber reformed. The SEE showed improved electrical responses in the right eye. The papilledema in the right eye gradually resolved (Figures 2b-2d).

One month later, blood tests revealed normalization of vitamin A and zinc levels (242 IU/dL and 114 μg/dL, respectively) and improvement in other vitamin deficiencies. The patient was discharged from the hospital. However, ten days after discharge, the left cornea perforated, possibly due to accidental scratching. Fortunately, the iris adhered to the cornea, and the corneal epithelium regenerated within a few days. She was managed conservatively with protective glasses and close monitoring.

Eleven months after the initial presentation, the BCVA improved to 0.7 in the right eye. However, vision in the left eye remained limited to light perception due to an irreversible corneal staphyloma and scar. The patient continued to have an unbalanced diet and required ongoing tube feeding at home due to insufficient oral intake.

Case 2

A 13-year-old boy with ASD and an intellectual disability was referred to our hospital by a pediatrician due to right corneal opacity and difficulty opening his eyelids. Five months prior to presentation, he developed facial nerve palsy and received oral adrenal corticosteroid treatment. Additionally, he experienced hyperemia and corneal opacity in the right eye, which did not improve with the use of antibiotic eye drops and ointment.

His medical history included bladder stones and buttock abscesses. He had a history of being a picky eater, mostly consuming white rice and noodles. He occasionally ate meat and fish but rarely and did not consume vegetables. His height was 140.3 cm (-2.47 SD) and his weight was 37.2 kg (-1.47 SD).

**Figure 5:**
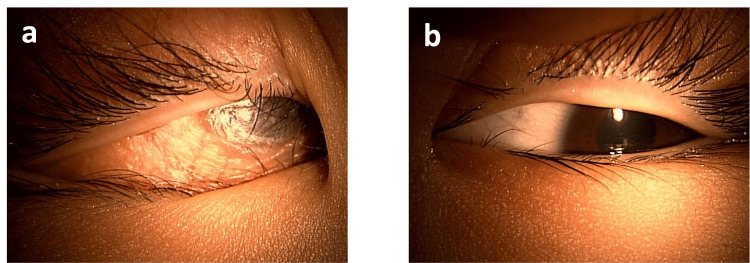
Anterior segment findings at initial examination a. Slit-lamp examination revealed marked dryness, conjunctival, and corneal keratosis in the right eye. b. The left eye appeared normal.

A slit-lamp examination revealed decreased wetting, keratoconjunctival keratosis, and corneal opacity in the right eye (Figure 5). Patient cooperation was not obtained for visual acuity or fundus examinations. Blood tests revealed that his serum vitamin A level had decreased to 6 IU/dL. Prealbumin was 16.1 mg/dL. 25-hydroxy-vitamin D was 16.1 ng/mL. Zinc was 65 μg/dL. Selenium was 9.3 μg/dL.

**Figure 6:**
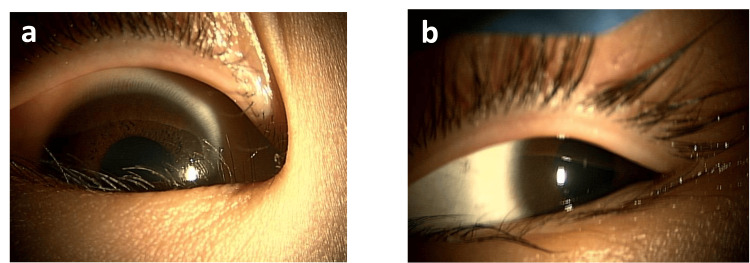
Anterior segment finding after oral administration of multivitamins of case 2 a. Dryness and keratinization had improved and the cornea became clear in the right eye. b. The left eye remained normal.

Based on these findings and his dietary history, he was diagnosed with xerophthalmia due to VAD, and oral administration of multivitamins (containing 2500 IU of vitamin A) and zinc acetate supplementation were initiated. From the fourth day of oral intake, improvements in dryness and keratinization of the right eye were observed. Corneal transparency was observed after approximately 20 days (Figure 6). The patient continued to take oral multivitamins, and no recurrence was observed after 11 months.

## Discussion

The first case demonstrates the potential for severe complications, including optic disc edema and irreversible corneal damage. The second case, fortunately, identified and treated early based on learning from the first case, demonstrated the importance of early intervention. 

Although VAD is often considered a problem in developing countries, children with ASD in developed nations like Japan are also at risk of VAD due to diets associated with the disorder. The rising prevalence of ASD, currently affecting an estimated 1 in 100 children [6], necessitates increased awareness of this potential health issue.

Children with ASD frequently exhibit dietary imbalances, often omitting vegetables, fruits, and protein sources in favor of processed foods and starches [7]. This pattern can lead to various nutritional deficiencies, including vitamin A. Previous studies have shown lower serum vitamin A levels in children with ASD compared to typically developing children [8].

Difficulties in communication and examinations can significantly delay the proper diagnosis of VAD in children with ASD, potentially leading to poorer prognoses [9]. Studies in Japan have reported severe cases of xerophthalmia in ASD patients with unbalanced diets for two to three years. These patients often present with extensive corneal damage, even after improvement with vitamin A treatment. In some cases, the damage has been irreversible, leading to complications such as corneal perforation and enucleation [4,10].

VAD can contribute to optic neuropathy [11-13]. Two mechanisms have been proposed. First, VAD may stimulate osteoblasts, leading to hyperostosis around the optic nerve canal, squeezing the nerve, and causing ischemia and damage [11,12]. Godfrey et al. reported cases of children with ASD who had low vitamin A levels, hyperostosis, and optic neuropathy. MRI scans showed increased signal intensity in both optic nerves, and computed tomography scans revealed diffuse skull hypertrophy [12]. The second mechanism is increased intracranial pressure in hypovitaminosis. Dontan et al. observed this in children with VAD, who also had bilateral papilledema due to the pressure. These children also had poor visual acuity and a pale optic disc at the time of examination. Notably, lower serum vitamin A levels were linked to worse visual outcomes [13].

In case 1, papilledema was present, but there was no clear evidence of skull hyperostosis. While we could not confirm it due to the lack of spinal fluid analysis, increased intracranial pressure due to VAD could be a possible explanation for the papillary swelling. Additionally, deficiencies in folate and vitamin B12, which can also cause optic neuropathy, were observed. Although vitamin A levels were severely low in case 1, nutritional treatment was initiated before the optic disc became pale. This early intervention led to a reversal of the papillary edema within approximately 35 days. In VAD cases, examining the optic nerve is crucial to assess potential intracranial complications and identify any co-existing nutritional deficiencies. 　

While the recommended initial treatment for childhood xerophthalmia is a high dose of vitamin A (200,000 units daily for two days, followed by another dose two to four weeks later) [1], our cases responded well to a lower dose. In our patients, treatment included not only vitamin A but also supplementation for other identified nutritional deficiencies. Additionally, ongoing dietary guidance was provided. This multi-faceted approach likely contributed to the positive outcomes.

## Conclusions

Vitamin A deficiency, a common cause of blindness in the developing world, can also occur in developed countries such as Japan. Children with ASD are particularly at risk for VAD due to their tendency toward restricted diets and communication difficulties. To prevent irreversible vision loss, ophthalmologists must be aware of the signs of VAD in children with ASD. Difficulty opening the eyelids and rubbing the eyes, often overlooked symptoms, should raise suspicion of VAD in children with ASD.
